# Inferencing Skill and Attentional Control Account for the Connection Between Reading Comprehension and Mathematics

**DOI:** 10.3389/fpsyg.2021.709944

**Published:** 2021-10-08

**Authors:** Young-Suk Grace Kim

**Affiliations:** University of California, Irvine, Irvine, CA, United States

**Keywords:** mathematics, reading, inference, attentional control, vocabulary

## Abstract

We examined the relations of inference, vocabulary, decoding, short-term memory, and attentional control to reading comprehension and mathematics performance for first-grade students in the US (*N* = 83). The students were composed of 75% Hispanics, 15% Whites, and 6% Asian Americans. Students' performance on mathematics and reading comprehension were very strongly related (*r* = 0.88). Results from path analysis showed that inference (0.27 ≤ s ≤ 0.38) was independently and positively related to both reading comprehension and mathematics performance after accounting for short-term memory, attentional control, decoding, and vocabulary. Decoding was independently related to reading comprehension, but not mathematics, whereas vocabulary was independently related to mathematics, but not to reading comprehension. Attentional control was directly related to mathematics, and indirectly related to reading comprehension and mathematics via inference, vocabulary, and decoding, with a substantial total effect on reading comprehension and mathematics (0.56 respectively). Short-term memory was not directly nor indirectly related to reading comprehension and mathematics. Overall these results show that language and cognitive skills are shared resources of reading comprehension and mathematics, and highlight the roles of attentional control and inference skill in reading comprehension and mathematics.

## Introduction

By now, there is robust evidence that reading and mathematics skills are related. Studies have consistently shown moderate to fairly strong relations between reading and mathematics (Aunola et al., 2004; Duncan et al., 2007; Grimm, 2008; Vilenius-Tuohimaa et al., 2008; Hart et al., 2010; Bailey et al., 2014; Korpipää et al., 2017, 2019; Erbeli et al., 2020; Koponen et al., 2020; Rinne et al., 2020; Vanbinst et al., 2020). For example, word reading and mathematics performances were moderately related with correlations ranging from 0.44 to 0.55 for first graders (Bailey et al., 2014). Another study showed that reading (composed of word reading and reading comprehension) and mathematics skills had fairly strong relations with correlations ranging from 0.65 to 0.67 for 7-to 12-year-olds (Hart et al., 2010). A recent meta-analysis also showed that students who experience a mathematics disability are two times more likely to have a reading disability (Joyner and Wagner, 2020). In the present study, we investigated sources of the relation between reading and mathematics, using data from first graders in the US.

## Sources of the Relation Between Reading and Mathematics Skills

Extant literature suggests several sources for the shared variance between reading and mathematics skills, including domain-general cognitive skills such as working memory and attentional control, and oral language skills such as vocabulary. According to theoretical models of reading (Kim, 2020) and mathematics (e.g., Geary, 1993; Geary and Hoard, 2005), domain-general cognitive skills or executive functions such as working memory and attentional control are foundational for reading and mathematics, respectively. Reading and mathematics both rely on holding and processing visual, phonological, and semantic information, and encoding and retrieving this information (Dehaene and Cohen, 1995; Geary and Hoard, 2005; Kim, 2020), for which working memory and attentional control are necessary. Indeed, a large number of studies have shown that working memory is related to mathematics (e.g., Bull and Scerif, 2001; Alloway et al., 2005; Koponen et al., 2007, 2020; Willcutt et al., 2013; Fuchs et al., 2016; Wang et al., 2016; Korpipää et al., 2017, 2019; Caviola et al., 2020; Rinne et al., 2020) and reading (e.g., Barnes et al., 1996; Swanson and Howell, 2001; Cain et al., 2004; Kim, 2017, 2020; Kim et al., 2018; Peng et al., 2018). Studies also showed the relation of inhibitory and attentional control to mathematics (e.g., Bull and Scerif, 2001; Fuchs et al., 2005, 2006; Gold et al., 2013; Rinne et al., 2020) and reading (e.g., Conners, 2009; Arrington et al., 2014; Kim, 2020). In addition, sustained attention was associated with comorbidity of math and reading difficulties (Barnes et al., 2020).

Another widely recognized source of the relation between reading and mathematics is oral language skills. For word reading, phonological processing is essential for mapping phonological representations with orthographic representations (e.g., Adams, 1990; Wagner et al., 1997; National Reading Panel., 2000). For reading comprehension, one must understand the words in a text to construct propositions of the given text (Anderson and Freebody, 1979), and quality lexical representation of a word allows efficient access to semantic information and successful reading comprehension (Perfetti, 2007). Therefore, vocabulary knowledge is important to reading comprehension (e.g., National Reading Panel., 2000; Perfetti and Hart, 2002; Elleman et al., 2009; Quinn et al., 2020). However, vocabulary knowledge is not sufficient for comprehension. Discourse comprehension of oral texts, listening comprehension, is also needed for reading comprehension (Gough and Tunmer, 1986; Hoover and Gough, 1990; Florit and Cain, 2011; Joshi et al., 2012; Kim, 2017, 2020).

Oral language skills are also important to mathematics. Verbal code is necessary for the development of number concepts because it connects the visual Arabic number code with the magnitude representation code (Geary, 1993; Dehaene and Cohen, 1995). Furthermore, much of mathematical knowledge and problems inherently relies on oral language skills such as vocabulary (both general and math-specific vocabulary) and listening comprehension. Not surprisingly, a rich body of studies indicates the relations of oral language skills to mathematics, including phonological processing (Hecht et al., 2001; Swanson and Sachse-Lee, 2001; Durand et al., 2005; Simmons et al., 2008; LeFevre et al., 2010; Koponen et al., 2020; Vanbinst et al., 2020), vocabulary (Durand et al., 2005; Fuchs et al., 2006; LeFevre et al., 2010; Purpura et al., 2011; Hornburg et al., 2018; Rinne et al., 2020), and listening comprehension (Aunola et al., 2004; Durand et al., 2005; Willcutt et al., 2013; Wang et al., 2016). For example, children's vocabulary and phonological awareness in preschool and kindergarten predicted their early numeracy skills (i.e., number naming), and their language skill composed of phonological awareness, vocabulary, and rapid automatized naming consistently predicted conventional mathematics skills 2 years later (e.g., numeration, measurement, number line; LeFevre et al., 2010). In a study of co-occurrence between reading and mathematics difficulties, Willcutt et al. (2013) found that verbal comprehension composed of vocabulary and comprehension explained reading and mathematics difficulties.

Another important source of the relation between mathematics and reading—reading comprehension in particular—is reasoning. Reasoning has long been considered important for mathematics skill (Russell, 1919; Piaget, 1952). Perhaps not surprisingly, reasoning is one of the eight standards for mathematical practice in the Common Core State Standards for mathematics (National Governors Association Center for Best Practices Council of Chief State School Officers., 2010), which are widely adopted in US schools. Reasoning is a broad, multi-dimensional, higher order construct that taps inferential skills, and includes deductive, inductive, causal, visual/spatial or non-verbal, and verbal reasoning. Studies have investigated and shown the roles of deductive, inductive, and non-verbal reasoning in mathematics skills (e.g., Handley et al., 2004; Cowan et al., 2005; Fuchs et al., 2005, 2016; Inglis and Simpson, 2008, 2009; Barkl et al., 2012; Morsanyi et al., 2013, 2017; Davidse et al., 2014; Wang et al., 2016).

Reasoning is also crucial for reading comprehension. Reading comprehension involves constructing propositions and integrating them to build a coherent mental representation of the text called the situation model (Kintsch, 1988). The text does not always explicitly provide all the information necessary for successful comprehension. Therefore, it is important for readers to make inferences to fill in the gaps, integrate information in the text, and integrate information in the text with prior knowledge (Kintsch, 1988; McNamara and Magliano, 2009). A rich body of studies has shown that inference skill is important to reading comprehension (e.g., Yuill and Oakhill, 1988; Barnes et al., 1996; Cain and Oakhill, 1999; Cain et al., 2004; Kim, 2020). Cain et al. (2004) showed that children's inferencing skill was related to reading comprehension after controlling for word reading, vocabulary, and working memory. Inference was also related to reading comprehension after accounting for working memory, attentional control, vocabulary, grammatical knowledge, comprehension monitoring, and perspective taking (Kim, 2020). Furthermore, poor comprehenders differed from their age-and skill-matched peers in their inferencing skill (Cain and Oakhill, 1999).

## Present Study

Previous studies indicated that language and cognitive skills make contributions to both reading and mathematics skills. In the present study, we build on and expand prior work by investigating the relations of oral language (vocabulary), domain-general cognitions (working memory and attentional control), decoding, and inference to reading comprehension and mathematics for students in Grade 1. The question that guided the present study was as follows: How are working memory, attentional control, vocabulary, decoding, and inference related to reading comprehension and mathematics for students in Grade 1?

Note that short-term memory was included as part of working memory (e.g., Davidson et al., 2006). We hypothesized that all the included skills would be related to reading comprehension and mathematics based on prior evidence. The role of decoding in reading comprehension is well-established (Gough and Tunmer, 1986; Hoover and Gough, 1990; Florit and Cain, 2011). Although previous studies did not focus on the role of decoding in mathematics, we hypothesized its role as decoding is necessary for mathematics tasks that include written texts beyond numerals.

Of the language and cognitive skills, we were particularly interested in the role of inference to reading comprehension and mathematics over and above the other skills. As stated above, evidence from the reading literature and mathematics literature, respectively, clearly indicates that reasoning is important to both reading comprehension and mathematics. However, slightly different aspects of reasoning were investigated in reading and mathematics fields, respectively. In mathematics, prior investigations focused on inductive reasoning (Barkl et al., 2012), transitive deductive reasoning (e.g., Handley et al., 2004; Morsanyi et al., 2013, 2017), and conditional deductive reasoning (e.g., Inglis and Simpson, 2008, 2009). In reading, prior investigations focused on causal inference such as making inferences using prior knowledge (i.e., elaborative inference) or making inferences using information in the text (i.e., bridging inference). In this study, we investigated whether students' elaborative inference skill is related to mathematics as well as reading comprehension. Elaborative inference captures skill in inferring information and relations using explicitly stated or provided information and extrapolating beyond the information provided. As such, underlying causal elaborative inference, and deductive and inductive reasoning are inferential processes, and therefore, elaborative inference skill would be relevant to various dimensions of mathematics (e.g., estimation, numeration, computation, word problems).

## Method

### Participants

The sample included 83 students in Grade 1 (55% females; *M*_*age*_ = 6.83) from eight classrooms in four schools in the Southwestern part of the US. The sample was composed of 75% Hispanics, 15% Whites, and 6% Asian Americans. All children in the participating classrooms were invited, and only consented children were included. The only exclusion criterion was students with identified intellectual disabilities, but no consented students were excluded based on this criterion. ~67% of the students were eligible for the free and reduced lunch program, a proxy for poverty. ~52% of students were classified as English learners (or limited English proficiency) according to the school district records.

### Measures

Students were assessed on reading comprehension, mathematics, inference, vocabulary, decoding, short-term memory, and attentional control. Unless otherwise noted, all the items were scored dichotomously, and reliability estimates are from the present sample. Reliability estimates were good to excellent and are reported in Table 1. Any questions from students regarding the task were addressed in the beginning of each task where the task was explained, and practice items were provided.

**Table 1:** Descriptive statistics.

**Variable**	**Reliability**	**Mean**	**SD**	**Min–Max**	**Skewness**	**Kurtosis**
MAP reading SS	0.97^+^	150.58	13.16	118**–**194	−0.04	1.15
MAP reading percentile rank	NA	32.12	24.48	1**–**99	0.67	−0.16
MAP math SS	0.97^+^	152.44	15.92	121**–**216	0.58	2.03
MAP math percentile rank	NA	33.35	28.36	1**–**99	0.62	−0.73
CASL-2 inference raw	0.93	12.15	6.98	0**–**22	−0.58	−1.07
CASL-2 inference SS	NA	83.01	16.15	54**–**125	−0.42	−0.70
CELF-4 vocabulary raw	0.87	17.05	9.57	2**–**44	0.36	−0.58
CELF-4 vocabulary SS	NA	5.63	3.34	1**–**15	0.42	−0.49
TOWRE-2 decoding raw	0.92^++^	9.74	8.81	0**–**52	2.43	8.43
TOWRE-2 decoding SS	NA	91.00	13.93	68**–**145	1.43	3.83
CTOPP-2 digit Span raw	0.88	11.31	4.36	0**–**18	−1.55	2.08
CTOPP-2 digit Span SS	NA	6.58	2.86	1**–**12	−0.39	−0.38
SWAN attentional control	0.98	26.83	11.69	3**–**54	0.32	−0.31

+ *Northwest Evaluation Association, 2011;*

++ *Torgesen et al. (2012). MAP = Measures of Academic Progress; SS = Standard Score; CASL-2 = Comprehensive Assessment of Spoken Lanauge-2nd Edition; CELF-4 = Clinical Evaluation of Language Fundamentals-4th Edition; TOWRE-2 decoding = Phonological Decoding Efficiency subtask of the Test of Word Reading Efficiency-2nd Edition; CTOPP-2 = Comprehensive Test of Phonological Processing-2; SWAN = Strengths and Weaknesses of ADHD Symptoms and Normal Behavior Scale*.

#### Reading Comprehension

A standardized, nationally normed measure, the Reading task of the Measures of Academic Progress (MAP; Northwest Evaluation Association [NWEA], 2019) was used. MAP reading comprehension is a computer-adaptive, multiple-choice test. Students read literary and informational texts and answered questions about them; for vocabulary items, students also matched sentences to pictures or diagrams.

#### Mathematics

A standardized, nationally normed measure, the Mathematics task of Measures of Academic Progress (MAP, North West Evaluation Association [NWEA], 2011) was used. Like the reading task, MAP mathematics is a computer-adaptive, multiple-choice test. The items assessed students' understanding of place value, counting, cardinality, number and operations, representing and solving problems, and representing and interpreting data (Northwest Evaluation Association, 2011).

#### Inference

The Inference subtask of the Comprehensive Assessment of Spoken Lanauge-2nd Edition (CASL-2; Carrow-Woolfolk, 2017) was used. In this task, the student was presented with a brief scenario, then asked a question that required inference to answer correctly. For instance, the student heard “*Mandy wanted to wear last year's dress to school 1 day, but when she tried it on, she could not wear it. Why?”* The correct responses must reference the fact that Mandy has grown or the dress does not fit anymore. There were two practice items.

#### Vocabulary

The Inference subtask of the Clinical Evaluation of Language Fundamentals-4th Edition (CELF-4; Semel et al., 2003) was used. In this task, the student was shown illustrations of people, objects, and actions, and was asked to name them. There was one demonstration item (demonstrating naming of a pictured object) and two practice items.

#### Decoding

The Phonological Decoding Efficiency subtask of the Test of Word Reading Efficiency-2nd Edition (TOWRE-2; Torgesen et al., 2012) was used. In this task, the student was asked to read a list of words, which were listed in order of increasing difficulty, within 45 seconds. The number of correctly read words within the time was their score. Practice included reading aloud eight words.

#### Short-Term Memory

The Digit Span subtask of the Comprehensive Test of Phonological Processing-2 (CTOPP-2; Wagner et al., 2013) was used. In this task, the student was presented with a sequence of digits and had to correctly recall the given sequence. Sequences increased in length, and administration discontinued after three consecutive incorrect responses. Correct answers were provided to students for Items one to four, following the protocols of CTOPP-2.

#### Attentional Control

The Strengths and Weaknesses of ADHD Symptoms and Normal Behavior Scale (SWAN; Swanson et al., 2012) was used. SWAN is a behavioral checklist that includes 30 items rated on a seven-point scale, ranging from a score of one (*far below average*) to seven (*far above average*) to allow for ratings of relative strengths (above average) as well as weaknesses (below average). In the present study, we used the first nine items (e.g., “sustain attention on tasks or play activities,” and “follow through on instructions and finish school work/chores.”), which were shown to capture the respondent's ability to regulate attention (Sáez et al., 2012). Higher scores represent greater attentional control. Participating students' teachers completed the SWAN checklist.

### Procedures

The measures were administered individually in a quiet space in the schools. The order of assessment was as follows: short-term memory, vocabulary, inference, and decoding, which were administered ~1 week apart by trained research assistants. MAP Reading and Mathematics tasks were administered by teachers as part of district practices. SWAN and MAP tasks administration intervals varied depending on teachers.

## Results

### Descriptive Statistics

Table 1 shows descriptive statistics. The sample students' mean performances on the MAP Reading and Mathematics tasks were in the low average range compared to the norm sample. Similar low average performance was found in the CASL-2 Inference task. The mean standard score of the TOWRE-2 decoding task was in the average range whereas mean standard scores on the CELF-4 Vocabulary and CTOPP-2 Digit Span tasks were in the low range. Note, however, these results should be taken with caution because many students in the sample were English learners and these tasks were not normed for English learners. What is important for the analysis in this study is that there was sufficient variability among students in the measured skills, and distributional properties were all adequate.

Table 2 shows bivariate correlations. Reading comprehension and mathematics were very strongly related (*r* = 0.88). Inference, vocabulary, decoding, and attentional control were moderately to fairly strongly related to reading comprehension and mathematics (0.50 ≤ *r*s ≤ 0.64) whereas short-term memory was weakly related to reading comprehension and mathematics (0.22 ≤ *r*s ≤ 0.23).

**Table 2:** Correlations between measures.

	**1**	**2**	**3**	**4**	**5**	**6**
1. MAP reading comprehension	–					
2. MAP mathematics	0.88	–				
3. CASL-2 inference	0.55	0.64	–			
4. CELF-4 vocabulary	0.59	0.64	0.50	–		
5. TOWRE-2 decoding	0.58	0.50	0.29	0.44	–	
6. CTOPP digit span	0.23	0.22	0.27	0.20+	0.11+	–
7. Attentional control	0.58	0.59	0.42	0.51	0.57	0.11+

*All correlations are statistically significant (p < 0.05) unless marked by +. + p > 0.05. MAP = Measures of Academic Progress; SS = Standard Score; CASL-2 = Comprehensive Assessment of Spoken Lanauge-2nd Edition; CELF-4 = Clinical Evaluation of Language Fundamentals-4th Edition; TOWRE-2 decoding = Phonological Decoding Efficiency subtask of the Test of Word Reading Efficiency-2nd Edition; CTOPP-2 = Comprehensive Test of Phonological Processing-2; SWAN = Strengths and Weaknesses of ADHD Symptoms and Normal Behavior Scale*.

### Relations of Language and Cognitive Skills to Reading Comprehension and Mathematics

**Figure 1:**
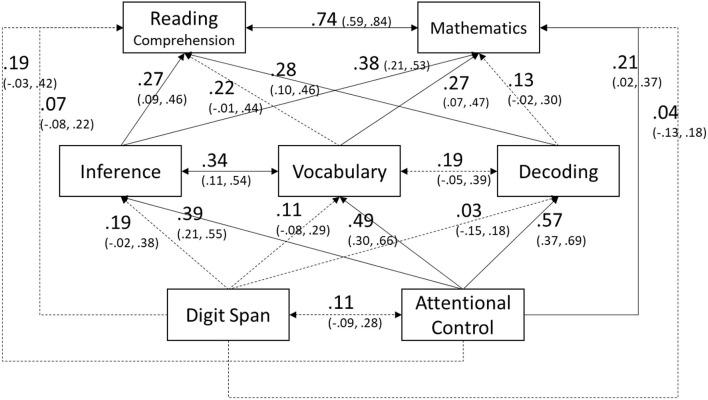
Standardized path coefficients (95% confidence intervals) where language and cognitive skills predict reading comprehension and mathematics. Solid lines represent statistically significant paths (*p* < 0.05) whereas dashed lines represent non-significant paths.

The path model shown in Figure 1 was fitted to the data using the maximum likelihood estimator, and model fit was excellent: χ^2^ (1) = 0.50, *p* = 0.48; CFI = 1.00; RMSEA = 0.00 [90% CI = 0.00, 0.26]; SRMR = 0.01. We used bootstrapping to estimate 95% confidence intervals. Standardized path coefficients and confidence intervals are shown in Figure 1. Reading comprehension was independently predicted by inference (0.27, *p* = 0.003) and decoding skill (0.28, *p* = 0.002). Vocabulary had a positive and statistically significant unique relation to reading comprehension when using a point estimate (0.22, *p* = 0.02), but confidence intervals included a zero and therefore was considered non-significant (see Figure 1). Mathematics was independently predicted by inference (0.38, *p* < 0.001), vocabulary (0.27, *p* = 0.002), and attentional control (0.21, *p* = 0.03). Attentional control was related to inference (0.39, *p* < 0.001), vocabulary (0.49, *p* < 0.001), and decoding (0.57, *p* < 0.001). The relation of attentional control to reading comprehension was marginally significant (0.19, *p* = 0.06) after controlling for short-term memory, decoding, vocabulary, and inference. Short-term memory was marginally related to inference after controlling for attentional control (0.19, *p* = 0.06). (Note that given the relatively small sample size, we have noted paths that were just shy of the conventional statistical significance of 0.05.) Indirect and total effects of attentional control and short-term memory were estimated. The indirect effects of attentional control on reading comprehension and mathematics were 0.37 (s.e. = 0.07, *p* < 0.001; 95% CI = 0.21, 0.53) and 0.36 (s.e. = 0.07, *p* < 0.001; 95% CI = 0.23, 0.50), respectively, and its total effects were 0.56 for both (95% CI for reading comprehension = 0.36, 0.71; 95% CI for mathematics = 0.38, 0.71). For short-term memory, indirect effect and total effect were 0.08 (s.e. = 0.05, *p* = 0.11; 95% CI = −0.01, 0.19) and 0.15 (s.e. = 0.09, *p* = 0.08; 95% CI = −0.04, 0.31), respectively, for reading comprehension, and 0.10 (s.e. = 0.06, *p* = 0.07; 95% CI = −0.01, 0.23) and 0.15 (s.e. = 0.09, *p* = 0.10; 95% CI = −0.07, 0.30), respectively, for mathematics. Approximately 55% and 60% of total variance in reading comprehension and mathematics, respectively, were explained by the included predictors.

## Discussion

In this study, we were interested in identifying sources of shared variance between reading comprehension and mathematics for students in Grade 1. Based on theory and prior evidence, we included language and cognitive skills, such as short-term memory, attentional control, decoding, vocabulary, and inference in our investigation.

Our findings revealed that inference was a common predictor of reading comprehension and mathematics for students in Grade 1 over and above short-term memory, attentional control, decoding, and vocabulary. Elaborative inference is part of a larger construct, reasoning, and is one of the necessary skills for establishing coherence and successful comprehension (Kintsch, 1988; Cain et al., 2004; Kim, 2020). Previous studies showed that different types of reasoning skills such as deductive and inductive reasoning and non-verbal reasoning skills contribute to mathematics (e.g., Handley et al., 2004; Cowan et al., 2005; Inglis and Simpson, 2009; Barkl et al., 2012; Morsanyi et al., 2013, 2017; Fuchs et al., 2016; Wang et al., 2016). In the present study, we used an inference task that requires students to infer information drawing on their background knowledge (i.e., elaborative inference). We hypothesized that elaborative inference would be important to mathematics because it captures one's skill in identifying and inferring relations, which is important to mathematical functions such as identifying and inferring patterns and relations (e.g., understanding how two or more items or numbers are related to each other) and deriving solutions. This hypothesis was supported as inference was independently related to both reading comprehension and mathematics even after accounting for the other language and cognitive skills. The results for reading comprehension are convergent with a large body of literature (e.g., Yuill and Oakhill, 1988; Cain and Oakhill, 1999; Cain et al., 2004; Kim, 2020) and theoretical models (e.g., van den Broek et al., 2005; Perfetti and Stafura, 2014; Kim, 2020). The findings for mathematics are in line with the importance of reasoning in mathematics performance. However, the relation of elaborative inference, a specific aspect of reasoning, to mathematics is novel in this study. These results suggest that primary grade students' skill in inferring unstated information using their background knowledge is a shared resource for reading comprehension and mathematics performance.

We also found that vocabulary was independently related to mathematics, but not to reading comprehension. Studies have shown that vocabulary knowledge, both general vocabulary knowledge and mathematical vocabulary words, is important to mathematics performance (Durand et al., 2005; LeFevre et al., 2010; Purpura et al., 2011; Hornburg et al., 2018; Rinne et al., 2020). The non-significant result for the unique relation of vocabulary to reading comprehension may appear inconsistent with theoretical models of reading (Perfetti and Stafura, 2014; Kim, 2020) and a large body of empirical evidence (e.g., Perfetti and Hart, 2002; Elleman et al., 2009; Quinn et al., 2020). However, the results are likely due to shared variance of vocabulary with inference (*r* = 0.50) and decoding (*r* = 0.44, see Table 2). The moderate relations of vocabulary with inference and decoding are in line with previous work (e.g., for inference, see Lepola et al., 2012; Tompkins et al., 2013; Currie and Cain, 2015; Kim, 2016, 2017; for decoding, see Ouellette, 2006; Ricketts et al., 2007). Vocabulary learning requires deriving or inferring meaning from context using meaning cues, and inferencing unstated meaning in a text relies on knowledge of vocabulary words (Currie and Cain, 2015; Kim, 2016). Furthermore, vocabulary knowledge is also hypothesized to be related to decoding via its relation with phonological awareness (e.g., Metsala, 1999) and irregular word reading (Ricketts et al., 2007). Therefore, the lack of an independent relation of vocabulary to reading comprehension over and above inference, decoding, short-term memory, and attentional control should not be taken as a lack of its contribution.

With regard to domain-general cognitions, attentional control and short-term memory, different patterns were found. Attentional control made a direct contribution to mathematics while it was marginally related to reading comprehension after controlling for the other skills. The relations of attentional control to reading comprehension and mathematics are in line with prior work (e.g., Bull and Scerif, 2001; Fuchs et al., 2005; Arrington et al., 2014; Barnes et al., 2020; Kim, 2020), and the present study extends prior work by showing the pathways of its contributions. That is, the present study revealed not only a direct relation of attentional control to mathematics and reading comprehension, but also the indirect relations of attentional control via inference and vocabulary (see Figure 1). In fact, indirect effects of attentional control on reading comprehension and mathematics were substantial. Studies have shown that attentional control is necessary for reading comprehension and mathematics (see above), and for vocabulary and inference skill (Saldert and Ahlsen, 2007; Smith et al., 2010; Nicolay and Poncelet, 2013; Kim, 2016, 2020), which contribute to reading comprehension and mathematics (see above). Therefore, it is important to recognize not only direct effects but also indirect effects of attentional control on reading comprehension and mathematics. This is in line with a recent theoretical model of reading, which explicitly articulated direct and indirect relations of skills to reading comprehension (Kim, 2020).

Unlike attentional control, short-term memory was not independently related to any of the predictors nor reading comprehension and mathematics. An exception is its marginally significant relation to inference. Note that short-term memory was related to reading comprehension, mathematics, and inference in the zero order correlations (Table 2), but it was not after accounting for attentional control. In other words, the present findings may be due to the moderate relation of attentional control to the other skills (0.42 ≤ *r*s ≤ 0.59, see Table 2) such that although short-term memory is related to inference, reading comprehension, and mathematics, it no longer has a unique relation over and above attentional control.

We found that decoding was uniquely related to reading comprehension but not mathematics. This is convergent with theoretical models of reading and a large body of evidence about the necessary role of decoding in reading comprehension (e.g., Hoover and Gough, 1990; Florit and Cain, 2011; Kim, 2017, 2020). Similar to the relation of vocabulary to reading comprehension, these results do not entail that decoding skill is not important for mathematics because decoding is necessary for any mathematics tasks that require students to read texts. What the present findings suggest is that although decoding skill was moderately related to mathematics (see Table 2), once the other predictors in the model were accounted for, it did not add a unique explanation of mathematics performance.

Taken together, these results indicate that the connection between early reading comprehension and mathematics is partly explained by and built on shared reliance on inferencing skill, general vocabulary knowledge, and attentional control. In other words, these are not specific to reading or mathematics performance. This implies that instruction on these skills would improve performance and development of reading comprehension *and* mathematics. Future experimental work is needed to test this hypothesis.

## Limitations and Future Directions

The generalizability of the present findings is limited to populations that share similar characteristics with the present sample, that is, first-grade students many of whom were English learners and from low socio-economic backgrounds. Theoretically the included language, cognitive, and decoding skills are expected to be important for students from various backgrounds, including L1 vs. L2 learners. However, the relative weight of their roles might differ as a function of language learner status (e.g., vocabulary may play a greater constraining role for L2 learners than for L1 learners; See Kim, 2020). Future replications with students from different demographic backgrounds are warranted. Furthermore, in this study, relations were estimated using observed variables which suffer from measurement error. Therefore, future replications are needed using latent variables for the included constructs.

Another important future direction is replication with a larger sample size. Given a relatively small sample size in the present study, some of the path coefficients in this study (i.e., short-term memory to inference; attentional control and vocabulary to reading comprehension) would have reached conventional statistical significance with a larger sample size. Despite this limitation, however, we believe the patterns found in the present study provide a good starting point for future exploration of shared language and cognitive sources of reading and math, and the nature of their relations.

Future studies should also explore other predictors of shared variance between reading comprehension and mathematics. For example, in the present study, we included elaborative inference, and future work can include other types of reasoning/inference skills such as deductive reasoning and non-verbal reasoning (Cowan et al., 2005; Pimperton and Nation, 2010) in conjunction with elaborative inference. This will reveal the relations among these different types of reasoning, and their shared and unique contributions to reading and mathematics.

Finally, the present study examined unidirectional relations, given cross-sectional data. However, bidirectional relations are hypothesized between language and cognitive (e.g., vocabulary) and reading comprehension according to theoretical models of reading (e.g., Kim, 2020). Such relations are also suggested between language and cognitive skills and mathematics (e.g., Cameron et al., 2019). Future longitudinal studies are warranted to investigate potential bidirectional relations.

## Data Availability Statement

The raw data supporting the conclusions of this article will be made available by the author upon request.

## Ethics Statement

The studies involving human participants were reviewed and approved by Human Subjects Research in the Office of Research in the University of California Irvine. Written informed consent to participate in this study was provided by the participants' legal guardian/next of kin.

## Author Contributions

Y-SGK conceived of the presented idea, collected and analysed data, and wrote the manuscript.

## Funding

This research was supported by the grants from the Institute of Education Sciences (IES), US Department of Education, R305A180055 and R305A200312, and National Institute of Child Health and Human Development (NICHD), P50HD052120.

## Author Disclaimer

The content is solely the responsibility of the author and does not necessarily represent the official views of the funding agencies.

## Conflict of Interest

The author declares that the research was conducted in the absence of any commercial or financial relationships that could be construed as a potential conflict of interest.

## Publisher's Note

All claims expressed in this article are solely those of the authors and do not necessarily represent those of their affiliated organizations, or those of the publisher, the editors and the reviewers. Any product that may be evaluated in this article, or claim that may be made by its manufacturer, is not guaranteed or endorsed by the publisher.
